# Etiology and clinical features of non-O1/non-O139 *Vibrio cholerae* infection in an inland city in China

**DOI:** 10.17305/bb.2022.8745

**Published:** 2023-08-01

**Authors:** Yu-Han Xiang, Qin-Qin Hu, Yan Liu, Rui Sheng, Jie Wang, Wen-Jing Li, Jian Shi, Xue Li, Shu-Hua Lu

**Affiliations:** 1 Clinical Laboratory, Affiliated Hospital of Jining Medical University, Jining, China; 2 Nursing Department, Affiliated Hospital of Jining Medical University, Jining, China; 3 School of Clinical Medicine, Jining Medical University, Jining, China

**Keywords:** Non-O1/non-O139 *Vibrio cholerae* (NOVC), *Vibrio cholerae*, infection, bacteremia, antimicrobial susceptibility

## Abstract

Non-O1/non-O139 *Vibrio cholerae* (NOVC) causes various illnesses ranging in severity from mild to life-threatening but were ignored previously. Knowledge of the NOVC infection, particularly bacteremia, is limited because of its rarity. Here, we retrospectively reported the demographic, clinical, and therapy characteristics of patients with NOVC infection. Isolated NOVC stains were identified by a series of biochemical, mass spectrometry, and serum agglutination tests. The results of 11 patients with NOVC infection (including eight with bacteremia) with a median age of 68 years were included in this report. Most isolated NOVC strains had antibiotic susceptibility. Patients with NOVC-positive were distributed in various departments, most occurring in gastroenterology (six cases). Hepatic disease was the most common comorbid disease, followed by diabetes (three cases) and biliary tract disease (three cases). Two cases were previously healthy. The most common symptom at presentation was fever. All patients presented with abnormal changes in hematology and inflammatory parameters. Cephalosporins were the most frequently used antibiotics. Ten patients had a favorable outcome after treatment; one died from complicated underlying diseases. In summary, we recommend the timely identification of NOVC strains using matrix-assisted laser desorption ionization-time-of-flight mass spectrometry (MALDI-TOF-MS). The suspicion of NOVC bacteremia cannot be ruled out regardless of the host’s immune status. An alternative therapeutic regimen for this infection may be β-lactam antibiotics or combined with β-lactamase inhibitors. Regardless, the specific therapeutic regimen should be based on the antibiogram data.

## Introduction

*Vibrio cholerae (V. cholerae)* is a pathogenic gram-negative bacillus autochthonous to the aquatic environment that infects humans through contaminated water or food [1]. Based on the lipopolysaccharide surface O-antigen, over 200 serogroups of *V. cholerae* exist [2]. *V. cholera* O1 and O139 are the two major serogroups responsible for epidemic cholera outbreaks worldwide [3]. In contrast, other *V. cholerae* serogroups, designated as non-O1/non-O139 *V. cholerae* (NOVC), are not associated with epidemic cholera [4]. NOVC are genetically diverse strains that are generally non-pathogenic in healthy hosts [5]. Therefore, the clinical significance of NOVC was ignored previously; until recently, when NOVC infections emerged in public discussions [6].

As emerging evidence shows, NOVC has several virulence genes (e.g., *toxR*, *hlyA,* and *ompU*) encoding the cholera toxin that synergistically contributes to the infection process [7, 8]. Therefore, NOVC could cause various illnesses ranging in severity from mild in immunocompetent patients to life-threatening in immunocompromised hosts [9]. Based on previous reports, NOVC infection frequently presents as hypothermia, hyperthermia, diarrhea, abdominal pain, and other presentations (e.g., abscesses, necrotizing fasciitis, cellulitis, and others) [10, 11]. In contrast, it sporadically presents as a rare or atypical clinical manifestation (e.g., bacteremia and epigastric pain) [12]. Although bacteremia caused by NOVC is rare among these infections, it almost invariably occurs in immunodeficient patients and is potentially fatal [13, 14]. NOVC bacteremia rarely occurs in healthy patients [12]. Because of its rare occurrence, the epidemiology, clinical manifestations, and pathogenesis of NOVC infection, particularly bacteremia, remain unclear [15]. Currently, existing knowledge on NOVC bacteremia is mainly from limited cases [16, 17]. Meanwhile, no large-scale trials have yet to be conducted; thus, recommendations regarding the choice of NOVC infection therapy are inconclusive [10]. Therefore, a comprehensive review of patients with NOVC infection will be valuable for developing early diagnosis and treatment.

NOVC infection is associated with exposure to seawater and seafood consumption [9]. In recent years, several emerging reports have also shown that NOVCs can grow in inland waters [18]. Mainland China, especially inland areas, rarely report NOVC infection cases [6]. Until 2020, only 35 NOVC infection cases were linked to inland water sources worldwide, as reviewed by Vezzulli et al. [19]. Here, we first report the demographic, clinical, epidemiological, and therapeutic characteristics of a patient cohort with NOVC infection in the inland city of Shandong, China, to contribute to the understanding and timely diagnoses of NOVC infection.

## Materials and methods

### Patients

We reviewed the clinical records of all patients diagnosed with NOVC infection from January 1, 2019, to December 31, 2021. A total of 11 cases (7 men, 4 women; median age, 68 [range, 37–73] years) with laboratory-confirmed NOVC infection were identified from three tertiary hospitals in Shandong, China. The demographic and clinical characteristics, including age, gender, disease history, clinical features, laboratory results, therapeutic regimen, and prognosis, were obtained from the medical records and entered into standardized data collection forms. If data were missing from the records or clarification was needed, we gathered data by direct communication with attending doctors. Two investigators (Yu-Han Xiang and Qin-Qin Hu) independently extracted and cross-checked the information for each case to ensure the accuracy of the data.

### Bacterial isolates and identification

NOVC strains were isolated from individuals with NOVC infection as per the following procedures. Blood, bile, pus, and stool samples were collected and processed in a microbiology laboratory within 30 min to isolate *V. cholerae*. Samples were ground under aseptic conditions and then inoculated on China blue agar plates (a selective medium similar to MacConkey agar [20]). China blue agar (Merck Millipore, Germany) is composed of meat extract (3 g/L), peptone from casein (5 g/L), sodium chloride (5 g/L), lactose (10 g/L), China-blue (0.375 g/L), and agar (12.0 g/L). An initial presumptive phenotype identification was performed according to colony morphology. The morphological characteristics of the isolated colonies were determined based on the color, shape, texture, size, and gram-staining of the colony. The hemolysis test was also performed to evaluate pathogenicity using a blood agar culture plate containing 4% Blood Agar Base (Merck Millipore, Germany) and 6% defibrinated goat blood. Thiosulfate-citrate bile salts-sucrose agar (TCBS) was used as the selective medium to isolate *V. cholerae*. Christie–Atkins–Munch–Peterson (CAMP) test was used to identify *V. cholerae* isolates by phenotype, using *Escherichia coli* (*E. coli,* ATCC25922) and *Staphylococcus aureus* (ATCC25923) as the quality control. CAMP-positive strains were assumed to belong to NOVC serogroups.

All isolates were subsequently examined by an automated Vitek-2 Compact system (Biomeriuex, France) and confirmed by matrix-assisted laser desorption ionization-time-of-flight mass spectrometry (MALDI-TOF-MS, BioMérieux, France). *E. coli* (ATCC8739) was used as the quality control for MALDI-TOF-MS. Meanwhile, the serotype of all isolates was confirmed by a serum agglutination test. *V. cholerae* strains that did not agglutinate with either O1 or O139 antisera were assumed to belong to NOVC serogroups.

### Antimicrobial susceptibility tests

Antimicrobial susceptibility of NOVC strains was determined using the AST-GN13 panel in the automated Vitek-2 Compact system following the recommendations of the Clinical and Laboratory Standards Institute (CLSI, 31st edition). Antibiotics were selected according to the first-line and second-line drugs used for NOVC infection, including amikacin, ampicillin, ampicillin/sulbactam, aztreonam, cefazolin, cefepime, cefotetan, ceftazidime, cefuroxime, cefoperazone/sulbactam, ciprofloxacin, gentamicin, imipenem, levofloxacin, meropenem, piperacillin, piperacillin/tazobactam, tobramycin, and sulfamethoxazole. The antibiotic susceptibility was judged as susceptible, intermediate, or resistant based on the breakpoints recommended in CLSI. *E. coli* (ATCC25922) and *S. aureus* (ATCC25923) were used as controls.

### Ethical statement

The study has been performed in accordance with the Declaration of Helsinki. The study protocol has been approved by the Institutional Review Board of the Affiliated Hospital of Jining Medical University (No. 2022C213). Informed consent was waived by the Institutional Review Board of the Affiliated Hospital of Jining Medical University due to the retrospective nature of the study.

## Results

### Phenotype and molecular identification and antimicrobial susceptibility of NOVC

**Figure 1.**
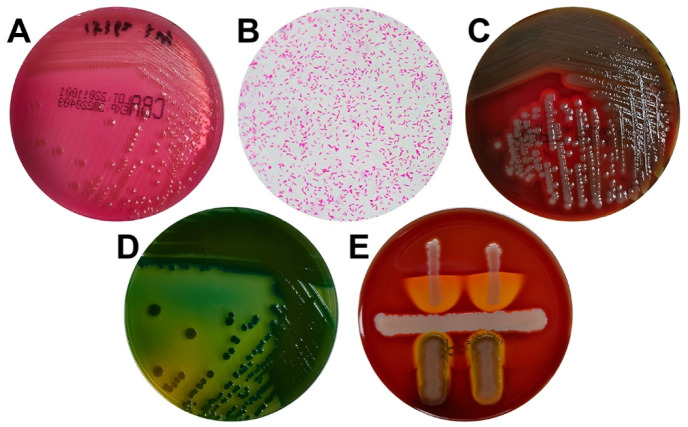
**Phenotype identification of *V. cholerae* strains.** (A) China blue agar showed small, round, tabular, and faint yellow colonies. (B) Gram stain showing Gram-negative curved bacilli (magnification, ×1000). (C) Blood agar showing β-hemolytic colonies. (D) TCBS agar presented as celadon (sucrose-fermenting) colonies. (E) CAMP test showed a crescent-shaped zone of complete synergistic hemolysis. CAMP: Christie–Atkins–Munch–Peterson test; TCBS: Thiosulfate-citrate bile salts-sucrose agar.

**Figure 2.**
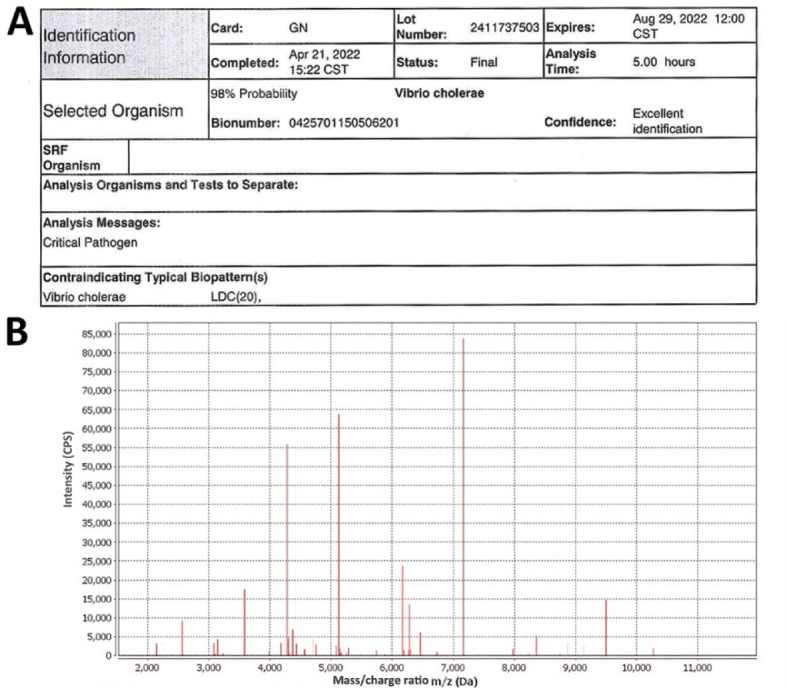
**Molecular identification of *V. cholerae* strains.** (A) Representative result of Vitek-2 Compact system analysis (case 5). (B) Representative result of MALDI-TOF-MS analysis. MALDI-TOF-MS: Matrix-assisted laser desorption ionization-time-of-flight mass spectrometry.

The phenotype identification of isolated colonies was determined by various media cultivations. The sample collected upon admission yielded small, round, tabular, and faint yellow colonies on the China blue agar plates (Figure 1A). Meanwhile, the blood culture grew gram-negative curved or straight bacilli (Figure 1B), which were non-lactose fermenting and showed β-hemolytic, oxidase-positive colonies on blood agar (Figure 1C). However, the culture on TCBS agar presented as celadon (sucrose-fermenting) colonies (Figure 1D). The CAMP test indicated a weakly positive result and appeared as a crescent-shaped zone of complete synergistic hemolysis (Figure 1E); thus, NOVC was suspected. The strains were then identified using an automated Vitek-2 Compact system. All strains had been routinely typed as *V. cholerae* using the Vitek-2 Compact system, with identification rates of 94%–98% (Figure 2A).

Further species confirmation was obtained by the MALDI-TOF-MS analysis, with confidence levels of 99.9% for all strains (Figure 2B). A serum agglutination test was performed to classify the serotype of the pathogen. The pathogen indicated self-coagulation in normal saline, gradually weakening after several passages. The strain displayed no agglutination with the O1 or O139 antisera. Thus, the strains were classified as NOVC. Antimicrobial susceptibility tests indicated that two strains were intermediate to ciprofloxacin, two were resistant to ampicillin and sulfamethoxazole, and the others were all sensitive to the tested antibiotics. Multidrug-resistant isolates were not detected.

### Demographic and clinical characteristics

Table 1 shows the demographic, clinical, and outcomes of all identified patients. Identified cases of NOVC infection included seven males and four females, with a median age of 68 years (range, 37–73 years). Patients with NOVC-positive were distributed in various departments, including gastroenterology (six cases), intensive care unit (two cases), infection (one case), intervention (one case), and gastrointestinal surgery (one case). Nine (81.8%) of 11 patients had at least one underlying comorbid disease. Hepatic disease (seven cases) was the most frequent comorbid disorder, followed by diabetes (three cases), biliary tract disease (three cases), hypertension (two cases), and one case each of pancreatic cancer, severe pneumonia, Behcet’s disease, bronchial asthma, and cerebral infarction. Only two cases were previously healthy. The most common symptoms at presentation were fever, fever with chills or rigors, edema of both lower limbs, and gastrointestinal symptoms, such as abdominal pain and diarrhea. According to the laboratory results, all patients presented with abnormal changes in hematology and inflammatory parameters (Table 2). Specifically, 7 (63.6%) of 11 patients indicated an increase in white blood cell counts, 10 (90.9%) an increase in neutrophil ratios, and 7 (63.6%) a decrease in platelet counts. In addition, platelet counts were elevated in two (18.2%) patients. Of nine patients who were evaluable for C-reactive protein levels (CRP), eight (88.9%) patients were CRP-positive (CRP ≥8 mg/L). The procalcitonin (PCT) levels were available in seven patients, and a positive PCT (PCT ≥0.5 ng/mL) was found in all seven (100%) patients. Only one patient had an increased level of hepatic function parameters.

**Table 1:** The demographic, clinical, and outcomes of all identified patients

**Cases**	**Age (years)**	**Sex**	**Underlying disease**	**Clinical features**	**Treatment regimens**	**Treatment course (days)**	**Outcomes**	**Samples**
Case 1	41	Male	Pancreatic cancer with liver metastasis	Fever	Ceftriaxone	6	Cured	Blood
Case 2	56	Male	Liver cirrhosis, hepatic encephalopathy, diabetes	Fever, dark stools, hematemesis	Ceftazidime	8	Cured	Blood
Case 3	70	Female	Autoimmune liver disease, biliary cirrhosis, hypertension	Fever, edema of both lower limbs	Imipenem/Cilastatin	5	Cured	Blood
Case 4	39	Male	Acute liver failure, severe pneumonia, hepatitis B, Behcet’s disease	Fever, edema of both lower limbs, skin icterus	Piperacillin/Tazobactam	6	Early withdrawal	Blood
Case 5	72	Male	Liver cancer, liver cirrhosis, hypertension, diabetes	Fever with chills or rigors, burning sensation in urination	Piperacillin/Tazobactam	14	Cured	Blood
Case 6	73	Female	Liver cirrhosis, ascites, diabetes	Fever, abdominal distension, diarrhea	Ceftriaxone	7	Cured	Blood
Case 7	69	Female	None	Abdominal pain, dizzy, headache, nausea	Sulbactam/Cefoperazone	3	Improved	Blood
Case 8	70	Female	Cholangiocarcinoma, bronchial asthma	Fever with chills or rigors	Sulbactam/Cefoperazone	9	Cured	Blood
Case 9	39	Male	Cholecystitis, common bile duct stones with cholangitis	Abdominal pain	Cefoxitin	4	Cured	Bile
Case 10	37	Male	None	Abdominal pain	Meloxicillin/Ornidazole	5	Cured	Pus
Case 11	68	Male	Liver cirrhosis, cerebral infarction, splenectomy and cholecystectomy	Fever, diarrhea	Ceftriaxone	5	Cured	Stool

**Table 2:** The laboratory results of all identified patients

**Cases**	**WBC (RefR: 3.5–9.5 ×10^9^/L)**	**Neutrophils (RefR: 40%–75%)**	**PLT (RefR: 125–350 ×10^9^/L)**	**CRP (RefR: <8 mg/L)**	**PCT (RefR: <0.05 ng/nl)**	**AST (RefR: 15–40 U/L)**	**ALT (RefR: 9–50 U/L)**	**GGT (RefR: 10–60 U/L)**
Case 1	9.96*↑*	79.70*↑*	84.00*↓*	NA	NA	40	33	57
Case 2	6.09	88.80*↑*	36.00*↓*	31.00*↑*	0.84*↑*	40	32	43
Case 3	10.78*↑*	86.50*↑*	44.00*↓*	50.00*↑*	0.52*↑*	38	34.6	19
Case 4	11.42*↑*	82.50*↑*	105.0*↓*	12.13*↑*	3.96*↑*	88*↑*	125.5*↑*	121*↑*
Case 5	8.73	91.70*↑*	64.00*↓*	67.36*↑*	3.04*↑*	31	32	70
Case 6	3.96	76.41*↑*	26*↓*	13.43*↑*	NA	32	33	52
Case 7	22.00*↑*	93.63*↑*	59*↓*	20.60*↑*	6.08*↑*	28	24	50
Case 8	12.00*↑*	81.67*↑*	415*↑*	109.40*↑*	0.56*↑*	29	25	24
Case 9	10.90*↑*	88.40*↑*	263	13.1*↑*	0.881*↑*	24	25.9	12
Case 10	16.15*↑*	86.94*↑*	352*↑*	NA	NA	20	18	19
Case 11	3.62	36.74	219	0.71	NA	18	24	20

WBC: White blood cell; RefR: Reference range; PLT: Platelet; CRP: C-reactive protein; PCT: Procalcitonin; AST: Aspartate aminotransferase; ALT: Alanine aminotransferase; GGT: Gamma-glutamyl transpeptidase; NA: Not available.

### Treatment and outcome

All 11 patients were treated with broad-spectrum antibiotics (Table 1). The most commonly used antibiotics were cephalosporins, such as ceftriaxone (cases 1, 6, and 11), ceftazidime (case 2), and cefoxitin (case 9). Five patients were empirically treated with a compound preparation of antibiotic and enzyme inhibitors, including imipenem/cilastatin (case 3), piperacillin/tazobactam (cases 4 and 5), and sulbactam/cefoperazone (cases 7 and 8). In addition, case 10 was synchronously given mezlocillin and ornidazole. Because of complicated underlying diseases, case 4 experienced multiple organ dysfunction syndromes and had to undergo mechanical ventilation but progressed to a critical condition. According to the decision of the patient’s family, the resuscitation attempt was refused. Another patient (case 7) showed improvement with a shorter 3-day treatment and was discharged according to the patient’s choice. The clinical symptoms of the remaining nine patients recovered after symptomatic treatment.

## Discussion

This is the first published study to provide a systematic description of the demographic, clinical, and therapy characteristics of 11 hospitalized patients with confirmed NOVC infection in an inland city in China, contributing to the understanding of NOVC infection.

The accurate and timely identification of NOVC is crucial for the early diagnosis of NOVC infection [10]. However, traditional biochemical methods are time-consuming, leading to a delayed diagnosis or underreporting NOVC. In this study, MALDI-TOF MS was used for the rapid identification of human-pathogenic *V. cholerae* to obtain consistent results with biochemical identification by the Vitek-2 Compact system. Similar to a previous report [21], MALDI-TOF MS is a robust tool for rapidly identifying Vibrio species. The minimal awareness among clinicians and the non-reporting of clinical cases might be another explanation for the underreporting of NOVC [12]. Although NOVC infections are sporadic, we still remind clinicians to be aware of the possibility of infections due to this pathogen.

Evidence shows that cirrhosis is the leading risk factor for NOVC bacteremia [9, 22]. Similarly, in our cohort, we observed that over half of the patients had hepatic disease. NOVCs are non-pathogenic in healthy hosts and rarely lead to fatal bacteremia [5]. Two healthy hosts were diagnosed with NOVC infection but recovered soon after short antimicrobial treatments. However, these cases were not alone, as previously reported by Deshayes et al. or Namdari et al. [10, 23]. Thus, it is recommended that the suspicion of bacteremia cannot be ruled out in immunocompetent hosts when NOVC infection.

NOVC is usually susceptible to most antimicrobial agents [9]. In the present study, we observed a similar phenomenon: over half of the strains were sensitive to the tested antibiotics. Several NOVC strains with antibiotic resistance have recently been described in environmental and clinical settings, involving trimethoprim, SXT [13], ampicillin [24], and even multidrug-resistant (MDR) strains [25]. Although the MDR strain was not detected in our cases, two strains were identified as ampicillin- and sulfamethoxazole-resistant, which was consistent with its known antimicrobial susceptibility pattern [26]. Thus, it is recommended that antimicrobial therapy in patients with NOVC infection must be based on antimicrobial susceptibility patterns, considering the heterogeneity of antibiotic resistance.

Based on the published cases of NOVC bacteremia, antimicrobial therapy demonstrated extreme heterogeneity in antibiotic type, dosage, and treatment duration [10]. In the present cohort, cephalosporin appeared to be the first-choice antibiotic and was associated with a favorable outcome, consistent with the previous experience [27, 28]. In addition, many of our cases received dual-agent therapy with antibiotic and enzyme inhibitors, especially in immunocompromised patients, as recommended by Couzigou et al. [29]. We noticed that treatment duration varies according to the patient’s background, clinical presentation, and severity, ranging from 3 to 14 days. Thus, the duration of treatment remains a matter of debate. Except for our one patient with complicated underlying diseases, all patients had a favorable outcome, regardless of the type, dosage, and treatment duration of antimicrobial agents. Based on the experience from our center, 1–2 weeks of β-lactam antibiotics or combined with β-lactamase inhibitors might be an alternative therapeutic regimen for this infection. Although recommendations regarding the choice of therapy are not conclusive, patients with NOVC infection, even bacteremia, usually have a favorable outcome in the current treatment pattern.

Several limitations of this study should be recognized. Firstly, a small number of patients were included due to the rarity of this disease. Secondly, the molecular characterization of NOVC strains might provide more support for NOVC identification, however, the lack of virulence gene profiles of NOVC was another limitation for this study since self-paying patients refused the gene testing. The virulence gene profiles of NOVC strains would be concerned in further studies. Thirdly, radiology results were not available due to the retrospective design, limiting the comprehensive description of this infection. Additionally, the lack of patient exposure history might limit the elaboration of infection transmission of NOVC in inland cities, so this needs further investigation.

## Conclusion

In this single-center study, we report for the first time on the largest cohort of patients with confirmed NOVC infection in an inland city in China. Although NOVC infections were sporadic, we still remind clinicians to be aware of the possibility of bacteremia, regardless of immunocompromised or immunocompetent individuals. MALDI-TOF MS is a robust tool for the rapid identification of this pathogen. In addition, β-lactam antibiotics combined with β-lactamase inhibitors may be an alternative therapeutic regimen for this infection. Regardless, the specific therapeutic regimen should be based on the antibiogram data.
